# Comparison of HbA1c and Time in Range in the Prediction of Large for Gestational Age in Pregnancies Involving Type 1 Diabetes

**DOI:** 10.3390/diagnostics16121900

**Published:** 2026-06-18

**Authors:** Katarzyna Rutkowska, Klaudia Czarnik, Katarzyna Cypryk

**Affiliations:** Department of Internal Diseases and Diabetology, Medical University of Lodz, Centralny Szpital Kliniczny, ul. Pomorska 251, 92-213 Lodz, Poland

**Keywords:** continuous glucose monitoring, neonatal outcomes, personal insulin pump, pregnancy, type 1 diabetes mellitus

## Abstract

**Background/Objectives**: While satisfactory glycaemic control is possible with specialist care from a diabetologist and modern therapies, women with type 1 diabetes are still subject to poorer obstetric outcomes, even with optimal management. **Methods**: The analysis comprised a cohort of 55 pregnant patients with type 1 diabetes who attended the Diabetology Outpatient Clinic between 2018 and 2023; all were recruited no later than the first trimester. Qualified patients underwent medical interviews and physical examinations. Insulin pump, continuous glucose monitoring (CGM) system, and postpartum data were collected. **Results**: The median glycated haemoglobin (HbA1c) at the beginning of pregnancy was 6.1%, with means of 5.9% and 6.0% in the following trimesters. Only 1/3 of the women achieved the recommended HbA1c value throughout pregnancy. The average/median time in range (TIR) in each trimester was ≤70%. Among the women who achieved the recommended TIR target, the infants tended to have lower birth weights but a higher likelihood of jaundice. Almost half of the newborns were large for gestational age (LGA), and a third were macrosomic. The strongest predictor of LGA was a mean blood glucose level > 124 mg/dL in the third trimester, which increased the risk of LGA by almost 12 times. **Conclusions**: Good diabetes control does not prevent LGA/macrosomia. TIR appears to be a better predictor of obstetric complications, including LGA. A mean glucose level ≥ 124 mg/dL in the third trimester greatly increases the risk of LGA.

## 1. Introduction

During pregnancy, the occurrence of diabetes significantly increases the risk of complications, which can include miscarriage, preterm delivery, foetal macrosomia and congenital anomalies [1,2].

As glucose is transferred across the placenta according to a concentration gradient, changes in maternal blood glucose levels have a direct effect on the foetus. As such, hyperglycaemia occurring prior to conception and during the first trimester has been associated with a greater risk of congenital malformations, foetal growth restriction and miscarriage.

In the foetus, pancreatic insulin production begins around week 10 of gestation, and it is secreted by pancreatic β-cells in response to glucose stimulation by week 20. During this period, maternal hyperglycaemia has been implicated in elevated foetal glucose concentrations and hyperinsulinemia, resulting in foetal tissue overgrowth. It also plays a role in various neonatal complications, including postnatal hypoglycaemia, respiratory distress syndrome and polycythaemia [3]. Among these, neonatal hyperinsulinemia and increased adiposity, driven by foetal insulin resistance, can result in a birth weight above the 90th percentile for gestational age [4]. The infants are also at increased risk of adverse long-term health outcomes, such as a higher prevalence of metabolic syndrome during childhood [5].

In women with pre-gestational diabetes, glycaemic control can be assessed by glycated haemoglobin (HbA1c) measurement. The American Diabetes Association recommends that target maternal glucose levels should be as close as safely possible to normal values [6]. Similarly, the Polish Diabetes Association (Polskie Towarzystwo Diabetologiczne, PTD) recommends target HbA1c levels of <6.5% during the preconception period and the first trimester, and <6.0% in subsequent trimesters [7].

It is recommended that pregnant women with type 1 diabetes mellitus (DM1) use continuous glucose monitoring (CGM) systems for glucose control, particularly integrated systems combining a high degree of automation with an insulin pump. Generally, CGM systems record interstitial glucose levels every five minutes, thus enabling real-time tracking and providing a detailed profile of glycaemic variability. This technology also measures other key parameters: time in range (TIR), i.e., the percentage of glucose readings within the recommended target range; time above range (TAR), i.e., the proportion of values exceeding the upper threshold; and time below range (TBR), representing episodes of hypoglycaemia.

During pregnancy, TAR, i.e., glucose levels above 140 mg/dL, should constitute less than 25% of daily measurements. TIR, i.e., the time spent with glucose levels between 63 and 140 mg/dL, should exceed 70% of measurements, while TBR, i.e., glucose levels below 63 mg/dL, should remain below 4% [7,8].

Therefore, the objective of the study was to determine the impact of metabolic testing (HbA1c and TIR) on obstetric outcomes and neonatal infant health in pregnant patients with type 1 diabetes mellitus, treated with personal insulin pumps and CGM. It also investigates the correlations between glycaemia measures and obstetric outcomes.

## 2. Materials and Methods

The study was approved by the Institutional Bioethics Committee of the Medical University of Lodz, Poland (RNN/334/20/KE and KE/563/23).

Initially, 64 women were recruited for the study, all of whom were attending the Diabetology Outpatient Clinic at the Central Clinical Hospital, Lodz, Poland between 2018 and 2023. All had been diagnosed with type 1 diabetes and were in the first twelve weeks of pregnancy.

All participants were receiving treatment with continuous subcutaneous insulin infusion (CSII) using a personal insulin pump with CGM, i.e., a MiniMed^TM^ 640G insulin pump, Guardian^TM^ Link 3 transmitter and Guardian^TM^ Sensor 3 (Medtronic MiniMed Inc., Warsaw, Poland). These devices include additional features aimed at improving glycaemic control, such as a bolus calculator and SmartGuard^TM^ technology. The devices were provided by the Wielka Orkiestra Świątecznej Pomocy charitable foundation.

Women with multiple pregnancies or who had conceived via in vitro fertilisation were excluded from the study, as were those diagnosed with neurological, psychiatric or cardiovascular diseases.

All participants received standard care in accordance with current national recommendations. They were counselled regarding target HbA1c values (less than 6.5% in the first trimester and less than 6.0% in the second and third trimesters) and recommended TIR goals (greater than 70% for glucose levels between 63 and 140 mg/dL) [7]. Throughout their pregnancies, the women remained under the care of both a diabetologist and a diabetes educator. Diabetes education is provided as a standard service under the NFZ (Polish national health fund) and consists of clinical topics such as treatment goals for diabetes in pregnancy, dietary recommendations, and physical activity guidelines, as well as technical training in CSII, glucometer and CGM use. During the first and second trimesters, intervals between visits did not exceed three weeks. In the third trimester, visits typically occurred every two weeks, increasing to every week in late pregnancy.

During the initial visit, a detailed medical history and anthropometric measurements were taken; laboratory tests and specialist consultations were also arranged in order to identify any chronic complications or comorbid conditions. The data from the insulin pumps and CGM systems were downloaded and compiled into reports using CareLink^TM^ (v3.1). Reports were generated from all available data for each trimester: the first trimester was assumed as the first 14 weeks, the second trimester from 15 to 28 weeks, and the third trimester from 28 weeks. Gestational age, trimester classification and due date were assumed based on the last menstrual period. Participants with sensor use of less than 70% were excluded from the study.

The medical histories of the recruited participants were reviewed. During the study, two participants were excluded due to miscarriage before 20 weeks of gestation, and seven others due to insufficient CGM data. Therefore, the final analysis included 55 women.

Gestational weight gain was defined as the difference between maternal pre-pregnancy weight (either self-reported or measured) and weight on the day of delivery. Maternal hypoglycaemia was defined as a glucose level of less than 63 mg/dL, and hyperglycaemia as greater than 140 mg/dL. The number of daily hypoglycaemic and hyperglycaemic episodes was assessed based on CareLink^TM^ reports.

Preterm birth was defined as delivery before the end of week 36 of gestation. Macrosomia was diagnosed when the birth weight exceeded 4000 g, and large-for-gestational-age (LGA) infants were defined as those with a birth weight above the 90th percentile for the Polish neonatal population [9]. Neonatal hypoglycaemia was diagnosed when the blood glucose concentration was below 40 mg/dL within the first 24 h of life. Neonatal jaundice was defined as a serum bilirubin level of >15 mg/dL and/or the need for phototherapy. Information on the occurrence of transient tachypnea of the newborn (TTN) was obtained from the newborn hospital discharge summary (i.e., diagnosis and/or clinical notes).

HbA1c levels were measured by high-performance liquid chromatography (HPLC) in each trimester of pregnancy. All tests were performed in the certified laboratory of the Central Clinical Hospital, Lodz, Poland.

Statistical analysis was performed using the Statistica 13.1 software (StatSoft Inc., Tulsa, OK, USA). The Shapiro–Wilk test was used to assess the normality of the distribution of the study variables. Continuous variables are presented as the mean and standard deviation (SD) or median and interquartile interval (25th and 75th percentile) according to their distribution. To compare groups with normally distributed data, a Student’s *t*-test was used. The Mann–Whitney U-test was used to compare groups with non-normally distributed data.

Univariate logistic regression models were built to analyse the correlation of mean glycaemia in each trimester with LGA. Receiver operating characteristic curve (ROC) analysis was used to establish an optimal cut-off point for mean glycaemia in the third trimester predicting LGA occurrence.

*p* values of less than 0.05 were considered statistically significant.

## 3. Results

The entire study cohort, comprising 55 pregnant women with DM1, was characterised and their glycaemic data was compared with their obstetric outcomes. The obtained data was then stratified by the degree of metabolic control, and intergroup comparisons were performed.

**Figure 1:**
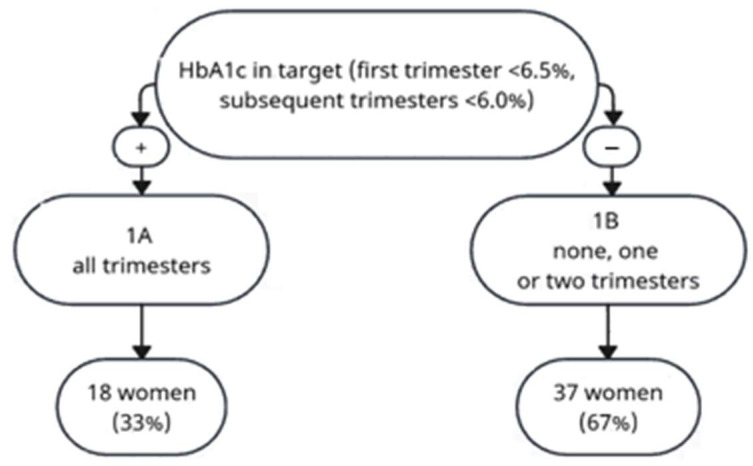
Grouping according to attainment of target HbA1c values, *n* = 55.

**Figure 2:**
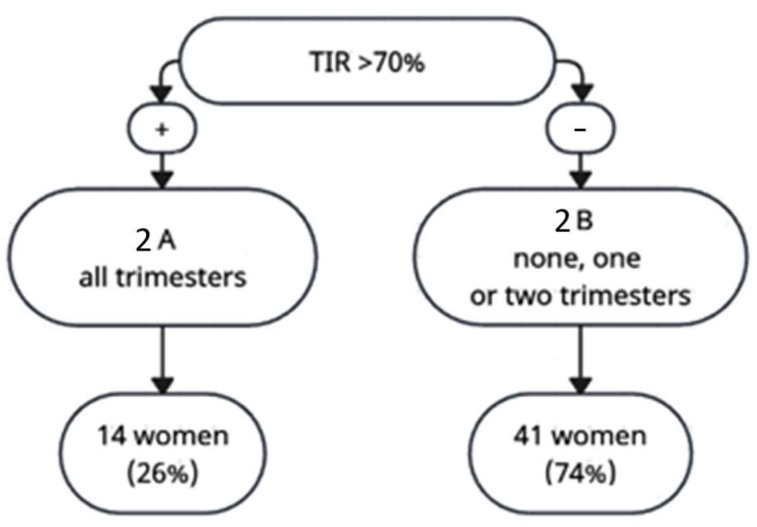
Grouping according to attainment of target TIR values, *n* = 55.

The cohort was divided into two groups (groups 1A and 1B) according to the number of trimesters in which they met the treatment goals based on HbA1c; only one-third of the women achieved target HbA1c throughout pregnancy (Figure 1). The entire cohort was also divided into groups 2A and 2B based on the number of trimesters in which they achieved the target TIR, defined as >70% of daily glucose measurements falling within the range of 63–140 mg/dL. This was achieved by slightly over one-quarter of participants throughout their pregnancies (Figure 2).

### 3.1. Characteristics of Study Participants

Baseline maternal characteristics and perinatal outcomes for the entire study group are presented in Table 1.

Glycaemic control improved over the course of pregnancy, as reflected in falling HbA1c and mean glucose values, and increasing TIR. The participants demonstrated good metabolic control in the early stages of pregnancy, with a median HbA1c level of 6.1% at conception; however, the mean HbA1c was 5.9% in the second trimester and 6.0% in the third. Mean sensor usage was 77% (trimester 1), 85% (trimester 2) and 87% (trimester 3). The TIR values were less than the recommended target in the first (mean = 68%) and second (mean = 69%) trimesters, but reached the target value (median = 70%) in the third. The total daily insulin requirement (per kg body weight) increased progressively during pregnancy, with a median increase of almost 43% between conception and delivery.

**Figure 3:**
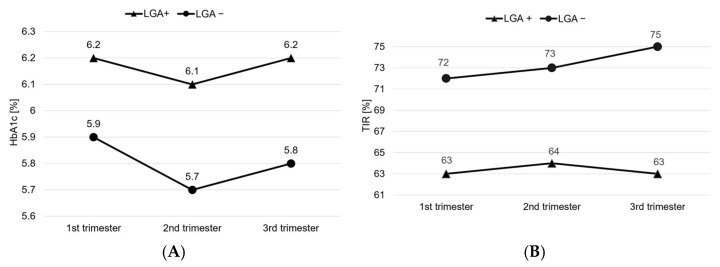
A comparison of HbA1c (**A**) and TIR (**B**) values between mothers of LGA and non-LGA newborns.

The mothers of LGA infants demonstrated significantly higher HbA1c and lower TIR than those of non-LGA infants for each trimester. The two groups also exhibited significantly different changes in TIR during pregnancy (Table 2; Figure 3).

### 3.2. Analysis by HbA1c Target Achievement

Groups 1A and 1B did not differ significantly with respect to age, duration of diabetes, pre-pregnancy weight, BMI, gestational weight gain, or the prevalence of diabetes complications.

Group 1A (i.e., well-controlled HbA1c) experienced significantly more frequent hypoglycaemic episodes and significantly fewer hyperglycaemic episodes in the first trimester, as well as lower mean sensor glucose values throughout pregnancy. They also had lower basal insulin requirements per kg of body weight throughout pregnancy compared to group 1B; this difference was significant in the third trimester (0.26 U/kg vs. 0.31 U/kg, *p* = 0.038). No intergroup differences were observed for other parameters.

Group 1A delivered LGA infants significantly less frequently than Group 1B (28% vs. 57%, *p* = 0.043). No significant intergroup differences were found regarding other obstetric outcomes.

### 3.3. Analysis by TIR Target Achievement

No significant differences were observed between groups 2A (i.e., well-controlled TIR for all trimesters) and 2B with respect to age, diabetes duration, pre-pregnancy weight, BMI, gestational weight gain, or the prevalence of diabetes complications.

**Figure 4:**
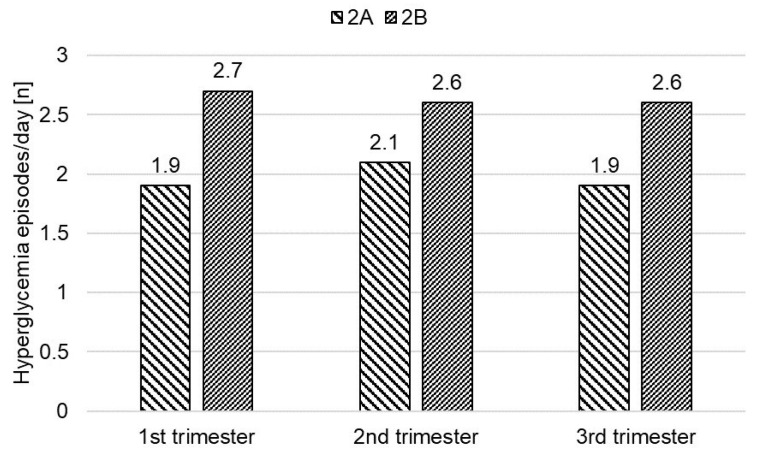
Mean hyperglycaemia episodes per day during pregnancy, depending on whether the target TIR values were achieved in all trimesters (2A) or not (2B).

Group 2A demonstrated significantly lower mean sensor glucose values for all trimesters and experienced hyperglycaemia far less frequently than Group 2B (Figure 4).

Obstetric outcomes for the groups are presented in Table 3. Maintaining a target TIR was associated with a significantly lower birth weight, a lower incidence of LGA, and a significantly higher rate of neonatal jaundice. However, this association was not explained by preterm birth.

### 3.4. Analysis of LGA Occurrence Relative to Glycaemic Control Parameters

The effect of various glycaemic control parameters, namely, pre-pregnancy weight, gestational weight gain, insulin requirements and the mean glucose level in each trimester, on the chance of LGA was determined by logistic regression. The findings indicate that LGA was significantly associated with the mean sensor glucose for each trimester and with changes in mean glucose levels throughout pregnancy.

The strongest predictor of LGA was the mean sensor glucose in the third trimester. Using ROC analysis, a cut-off value of 124 mg/dL was identified, above which the risk of delivering an LGA infant increased nearly 12-fold (OR [95% CI] = 11.81 [3.12–44.60], *p* = 0.0003). The relatively small sample size, particularly after subgroup stratification, should be considered when interpreting this result. The wide confidence interval indicates the limited precision of the effect estimate.

## 4. Discussion

Among pregnant women with diabetes, glycaemic control is typically assessed by self-monitoring of blood glucose levels, most commonly by determining the level of HbA1c: a well-established marker that reflects mean blood glucose levels over the preceding three months. While numerous studies have found HbA1c values to have a strong association with pregnancy outcomes [2,10,11], the value is not suitable for monitoring short-term fluctuations in glycaemia or assessing day-to-day glucose variability. Self-monitoring of blood glucose also provides only a limited view of overall glycaemic control.

The introduction of CGM systems has enabled improved metabolic control, thus improving maternal glycaemia, foetal metabolism and pregnancy outcomes, irrespective of the choice of insulin delivery method (i.e., multiple daily injections or continuous subcutaneous insulin infusion) [12,13]. For patients using CGM systems, an additional metric is TIR. The current TIR targets for pregnant women with DM1 were established in the 2019 international consensus document and have been adopted by numerous diabetes societies, including the Polish PTD [7,8].

The aim of the present study was to compare two methods of glycaemia evaluation, namely, traditional HbA1c-based evaluation and assessment including TIR, with regard to their potential to predict LGA in pregnant women with DM1. Both HbA1c and TIR values were recorded each trimester, together with their changes over the course of pregnancy. The mean HbA1c values in the first and second trimesters were found to be within the recommended national ranges, while the value in the third trimester was at the threshold. The change in HbA1c throughout pregnancy was −0.5%. Only 33% of participants achieved the recommended HbA1c target in each trimester, and 26% achieved the recommended TIR target. Only eight participants (15%) achieved complete glycaemic control, i.e., they met both HbA1c and TIR targets.

A subset of the participants included in the present study also took part in a multicentre Polish study, including almost 200 pregnant women with DM1 from 22 centres nationwide, published in January 2023 [14]. The study evaluated the impact of insulin pump therapy, with or without CGM, on glycaemic and obstetric outcomes. It was found that the mean HbA1c level decreased from 6.5% in the first trimester to 5.7% in the second trimester and 5.8% in the third. The percentage of time spent within the target glucose range (TIR value) was also comparable to our present findings. The two study groups demonstrated similar maternal age, pre-pregnancy BMI, and diabetes duration.

Another study including approximately 200 participants (221 in the first trimester and 172 in the third trimester) assessed the achievement of HbA1c and TIR targets in pregnant women with type 1 diabetes [15]. It identified higher HbA1c values each trimester (6.8%, 6.3%, and 6.3%) compared with the present study (6.1%, 5.9%, and 6.0%); however, this difference may be due to their exclusion of participants with a baseline HbA1c value of less than 6.5%. Their findings also indicate TIR values of 51.6% in early pregnancy, falling in the second trimester and then rising again to 64.1% in late pregnancy. Achieving the target HbA1c values was associated with a lower risk of LGA infants in each of the evaluated trimesters. Attaining the target TIR in the third trimester reduced the risk of preterm birth (*p* = 0.044), as did achieving the target HbA1c in the second and third trimesters (*p* = 0.002 and *p* = 0.005). In contrast, in the present study, no significant association was found between achieving either glycaemic target and the risk of preterm birth.

Maternal hyperglycaemia is known to be a cause of foetal overgrowth and related neonatal complications [1,2,16,17]. However, a cohort study of 102 pregnant women with type 1 diabetes reported a high prevalence of LGA births (27% of pregnancies), despite good glycaemic control [18]. Similar values were reported in the present study among the participants meeting glycaemic targets, with LGA rates of 28% in Group 1A and 21% in Group 2A. In contrast, however, a higher overall LGA rate (47%) was noted, with macrosomia present in 31% of newborns; this may be due to the lower proportion of women who achieved HbA1c (i.e., 33% vs. 59% noted in the cohort study) and TIR targets (i.e., 26% vs. 77% in the cohort study).

A previous study conducted in an identical population found that more than one-third of newborns experienced neonatal jaundice, and that their mothers tended to demonstrate significantly poorer second- and third-trimester TIR values than those without jaundice [19]. In contrast, the present findings indicate that achieving the target TIR (i.e., TIR >70%) was associated with a significantly higher incidence of neonatal jaundice. This unexpected association is difficult to interpret and should be considered with caution.

Several potential explanations may be considered. First, residual confounding cannot be excluded, as factors such as gestational age at delivery, mode of delivery, and maternal or neonatal characteristics may have influenced bilirubin metabolism and neonatal outcomes [20]. Second, differences in neonatal monitoring practices and bilirubin measurement protocols may have affected the detection rate of jaundice across subgroups [21]. Finally, given the relatively small subgroup size and multiple comparisons, a chance finding cannot be ruled out.

Therefore, while this association is noteworthy, it should be interpreted as hypothesis-generating and requires confirmation in larger, prospective studies with standardised neonatal assessment protocols.

Pregnancy complications are more common among diabetic mothers, even those with good glycaemic control. Even so, achieving normoglycemia during pregnancy remains crucial for optimising maternal and neonatal outcomes. Most studies examining the effects of glycaemic control base their analysis on traditional parameters, such as HbA1c, mean glucose and TIR.

A 1992 study found elevated maternal glucose levels in the third trimester to be strongly associated with macrosomia [22]. The study also noted that women with pre-gestational diabetes who gave birth to LGA infants tended to have significantly higher postprandial glucose values; the most pronounced difference was noted between 29 and 32 weeks of gestation: 145 ± 14 mg/dL for mothers with LGA infants compared to 137 ± 16 mg/dL for mothers with normal-sized infants (*p* < 0.005). The authors recommend a postprandial glucose target of less than 130 mg/dL to reduce the risk of macrosomia while lessening the chance of foetal growth restriction associated with maternal hypoglycaemia.

Similarly, a 2024 study proposed mean glucose concentrations of 122 mg/dL in the third trimester and 133 mg/dL in the second trimester as cut-off values for predicting LGA [23]. In the present study, a mean third-trimester glucose value over 124 mg/dL was associated with an approximately 12-fold increased risk of LGA (OR = 11.81; 95% CI: 3.12–44.60). However, the precision of this estimate should be interpreted with caution given the relatively small sample size, particularly after subgroup stratification into HbA1c- and TIR-based categories, which may have limited statistical power. The wide confidence interval further indicates substantial uncertainty regarding the exact magnitude of the observed association. This suggests that while the direction of the effect appears robust, the true effect size may be more variable and warrants confirmation in larger, adequately powered studies. This finding is also very close to the threshold of 126 mg/dL reported by Scott et al., who evaluated weekly CGM profiles during pregnancy [24]. Hence, it appears the currently accepted upper glucose target of 140 mg/dL may be too high.

Our findings support those of previous studies. They confirm that the use of CGM and other modern diabetes management tools can play a key role in reducing the incidence of LGA births. While HbA1c is of considerable value in retrospective assessment, reliance on HbA1c values alone is insufficient for optimal diabetes management during pregnancy. In contrast, TIR metrics allow better maternal and perinatal outcomes to be achieved, regardless of the treatment modality employed.

## 5. Strengths and Limitations

The primary limitation of this study is the relatively small sample size, which may reduce statistical power and increase uncertainty around the effect estimates. In addition, the single-centre design limits the external validity and generalisability of the findings. The inclusion of only participants with complete datasets may have introduced selection bias, as individuals with missing data could differ systematically in clinical characteristics and pregnancy outcomes compared with those included in the analysis. Furthermore, the possibility of unmeasured confounding factors cannot be excluded. Therefore, there is a need for further investigations based on larger cohorts to address these limitations.

Nevertheless, the study also has its strengths, one of which is the homogeneity of its study group. All participants were managed within a single, high-specialty tertiary care centre, which minimised the impact of potential confounding variables on the results obtained. The same self-management education, pre-pregnancy counselling and perinatal diabetes care models were used for all participants; the care was performed according to the same protocol and current PDA guidelines, and on the university premises at the Clinical Hospital. Furthermore, the studied glycaemic and metabolic parameters were assessed throughout pregnancy based on all available trimester-specific data.

Overall, while these findings should be interpreted with caution due to the above limitations, they may provide a useful basis for future research and contribute to the development of more detailed clinical recommendations for the management of women with type 1 diabetes during pregnancy.

## 6. Conclusions

Treatment with personal insulin pumps combined with CGM and self-monitoring achieved satisfactory glycaemic outcomes among pregnant women with diabetes under regular care at the studied Diabetology Outpatient Clinic. However, even optimal metabolic control does not fully eliminate the risk of neonatal complications, and achievement of target HbA1c values during pregnancy does not necessarily correspond to attainment of target TIR levels.

In this cohort, TIR appeared to be a more informative indicator of glycaemic control during pregnancy in relation to the risk of obstetric complications, particularly LGA. Exceeding a mean glucose level of 124 mg/dL in the third trimester was associated with an increased risk of delivering a macrosomic infant. However, given the retrospective, observational, single-centre design of the study, no causal inferences can be drawn, and the findings should be interpreted as hypothesis-generating. Further confirmation in larger, prospective, multicentre cohorts is required.

## Figures and Tables

**Table 1:** Baseline maternal characteristics and perinatal outcomes, *n* = 55.

Parameter	Value
Baseline maternal characteristics	
Age [years]	30.5 ± 3.9
Duration of diabetes [years]	13.9 ± 7.6
Pre-pregnancy body weight [kg]	68.8 ± 12.2
Pre-pregnancy BMI [kg/m^2^] *	24.0 (21.8; 27.1)
Retinopathy [*n*]	12 (22%)
Neuropathy [*n*]	1 (2%)
Nephropathy [*n*]	0 (0%)
Hypertension [*n*]	1 (2%)
Primiparous/Multiparous [*n*]	32 (58%)/23 (42%)
Gestational weight gain [kg]	11.6 ± 5.0
Pregnancy planning [*n*]	20 (36%)
Perinatal outcomes	
Birth weight [g]	3617 ± 671
Gestational age at delivery [weeks] *	38 (37; 39)
Preterm (<37 weeks)/term (≥37 weeks) delivery [*n*]	12 (22%)/43 (78%)
Vaginal delivery [*n*]/Caesarean section [*n*]	10 (18%)/45 (82%)
Sex (female/male) [*n*]	22 (40%)/33 (60%)
Macrosomia [*n*]	17 (31%)
LGA [*n*]	26 (47%)
Jaundice [*n*]	12 (22%)
Hypoglycaemia [*n*]	7 (13%)
Transient tachypnea of the newborn [*n*]	11 (20%)
Congenital anomalies [*n*]	0 (0%)

The results are presented as the number of cases [*n*], percentage [%], or mean ± standard deviation; An asterisk (*) indicates parameters without a normal distribution; these are presented as median and interquartile range (IQR).

**Table 2:** HbA1c and TIR in mothers of newborns with and without LGA.

Parameter	LGA + (*n* = 26)	LGA − (*n* = 29)	*p*, Value
HbA1c			
HbA1c in the 1st trimester [%] *	**6.2 (5.8; 7.1)**	**5.9 (5.7; 6.3)**	**0.048**
HbA1c in the 2nd trimester [%]	**6.1 ± 0.6**	**5.7 ± 0.5**	**0.033**
HbA1c in the 3rd trimester [%]	**6.2 ± 0.5**	**5.8 ± 0.4**	**0.010**
Change in HbA1c during pregnancy [percentage points] *	−0.5 (−0.7; −0.3)	−0.5 (−0.9; −0.2)	0.555
Change in HbA1c during pregnancy [%]	−9.3 ± 12.8	−7.4 ± 12.5	0.583
TIR			
TIR in the 1st trimester [%]	**63 ± 12**	**72 ± 12**	**0.021**
TIR in the 2nd trimester [%]	**64 ± 11**	**73 ± 11**	**0.007**
TIR in the 3rd trimester [%] *	**63 (53, 73)**	**75 (70; 82)**	**0.0003**
Change in TIR during pregnancy [percentage points]	**1 ± 11**	**12 ± 12**	**0.0004**
Change in TIR during pregnancy [%]	**2 ± 20**	**24 ± 26**	**0.002**

The results are presented as mean ± standard deviation; Parameters marked with an asterisk (*) do not have a normal distribution and are presented as median and interquartile range (IQR). Statistically significant results are in bold.

**Table 3:** Perinatal outcomes in the study groups: the TIR criterion.

Perinatal Outcomes	Group 2A (*n* = 14)	Group 2B (*n* = 41)	*p*, Value
Birth weight [g]	**3257 ± 717**	**3729 ± 623**	**0.026**
Gestational age at delivery [weeks] *	37 (37; 38)	38 (37; 39)	0.367
Preterm delivery (<37 weeks) [*n*]	4 (29%)	8 (20%)	0.479
Vaginal delivery [*n*]	14	20	0.662
Caesarean section [*n*]	86	80	0.662
Macrosomia [*n*]	3 (21%)	14 (34%)	0.374
LGA [*n*]	**3 (21%)**	**23 (56%)**	**0.007**
Jaundice [*n*]	**7 (50%)**	**5 (12%)**	**0.003**
Hypoglycaemia [*n*]	0 (0%)	7 (17%)	0.098
Respiratory distress syndrome [*n*]	3 (21%)	8 (20%)	0.877

The results are presented as the number of cases [*n*], percentage [%], or mean ± standard deviation; Parameters without a normal distribution are marked with an asterisk (*) and are presented as median and interquartile range (IQR). Statistically significant results are in bold.

## Data Availability

The data that support the findings of this study are available on request from the corresponding author, KR.
